# From Global Standards to Local Action: Practical Pathways for Adopting the International Council of Nurses’s (ICN's) Updated “Nursing” and “a Nurse” Definitions in Low- and Middle-Income Countries

**DOI:** 10.1177/15271544251408441

**Published:** 2025-12-26

**Authors:** Animesh Ghimire, Mamata Sharma Neupane

**Affiliations:** 1 School of Nursing and Midwifery, Faculty of Medicine, Nursing and Health Science, 2541 Monash University, Clayton, VIC, Australia; 2 Sustainable Prosperity Initiative Nepal, Baneshwor-31, Kathmandu, Nepal; 3 School of Nursing and School of Public Health, 475236 Chitwan Medical College, Bharatpur-5, Kailashnagar, Chitwan, Nepal

**Keywords:** ICN definitions, nursing regulation, LMICs, knowledge pluralism, workforce mobility, gender-equitable financing, planetary health, nursing

## Abstract

In 2025, the International Council of Nurses (ICN) issued renewed definitions of “nursing” and “a nurse,” marking the first comprehensive revision of its core terminology in more than two decades. The updated definition separates the discipline of nursing from the professional role of the nurse and adopts a rights-based, science-anchored, and climate-responsive framing. While early commentary from high-income countries has been largely positive, the implications for low- and middle-income countries (LMICs)—which carry the greatest share of the global nursing shortfall and depend heavily on community-based cadres—are more complex. This commentary critically examines the implications of ICN's updated definitions for LMIC settings. Drawing on a targeted review of MEDLINE, CINAHL, Scopus, and key ICN and World Health Organization documents, and guided by Walt and Gilson's policy analysis triangle, the paper analyzes how the definitions interact with context, actors, and processes in resource-constrained health systems. Three inter-related domains are explored: knowledge pluralism, financing, and workforce mobility. The analysis highlights how the definitions can elevate nursing's professional status, legitimize nurse leadership, and clarify regulatory expectations, while also risking the marginalization of unlicensed cadres and accelerating outward migration if adopted without safeguards. To translate global standards into equitable local practice, the paper outlines practical pathways for country-level integration: (1) establishing reciprocal evidence panels that combine culturally grounded and scientific knowledge in national guidelines; (2) study serve financing compacts that tie tuition relief to service in underserved regions, thereby converting the “triple dividend” of nursing investment into measurable returns; and (3) circular skills agreements that link international recruitment to reinvestment in domestic training capacity. Ultimately, the renewed definitions are a catalytic scaffold, not a panacea; their transformative potential will be realized only through deliberate, context-sensitive governance that aligns global standards with local realities.

## Introduction

In June 2025, the International Council of Nurses (ICN) released *Renewing the Definitions of “Nursing” and “a Nurse,”* the organization's first full rearticulation of professional terminology since 2002 (White et al., 2025). Definitions are not mere semantics: they regulate licensure, shape curricula, guide workforce planning, and influence cross-border credential recognition and signal the profession's claims to authority and responsibility in health systems. ICN's new text—rights-based, science-anchored, and environmentally responsive—separates, for the first time, the discipline (*nursing*) from the professional practitioner (*a nurse*) (White et al., 2025). High-income countries (HICs) hail the document as a long-overdue modernization, citing its transparent five-step development process, which included a multilingual survey, a five-round Delphi process, and consultation with National Nursing Associations. Importantly, this Delphi group included participants from several low- and middle-income countries (LMICs; Eswatini, the Philippines, Rwanda, and India), as well as an Indigenous nurse scientist from the United States (a member of the A’aninin Nation) whose research focuses on health equity in Indigenous communities.

Nevertheless, the implications are less straightforward for LMICs, which carry approximately 89% of the projected global nursing shortfall yet rely heavily on community health workers, auxiliary nurses, and other cadres whose titles fall outside ICN's binary of nurse/nonnurse (WHO, 2022). In these settings, the renewed definitions are a double-edged sword: they offer a powerful advocacy lever to elevate professional nursing standards and, at the same time, risk fragmenting the frontline workforce on which basic service delivery depends. To underpin this practice update, we undertook a purposive search of MEDLINE, CINAHL, and Scopus (January 2000–July 2025) using combinations of the terms “International Council of Nurses,” “definition of nursing,” “nurse regulation,” “low- and middle-income countries,” “workforce mobility,” and “planetary health,” supplemented by targeted searches of ICN and World Health Organization (WHO) policy documents. Analytically, the paper is informed by Walt and Gilson's policy analysis triangle, which examines how content, context, actors, and processes interact in policy change (Walt & Gilson, 1994), and by implementation-science perspectives on how global norms are translated into local practice (Nilsen, 2015). This article interrogates the dual character of the new definitions by (a) tracing the historical evolution of ICN terminology, (b) critiquing the 2025 report's methodology and assumptions, and (c) analyzing potential gains and pitfalls for resource-constrained health systems. The goal is not to dismiss ICN's achievement, nor to imply that the definitions were intended as a panacea, but to map the governance, regulatory, and financing work required to translate a global standard into equitable, context-specific practice in LMICs.

## A Redefined Profession: Brief Review and Discussion

### Evolution of the ICN Definitions

The 2025 pair of definitions retains nursing's holistic ethos yet introduces four pillars with direct implications for LMIC policy: (1) explicit human-rights language, (2) cultural safety informed by Indigenous scholarship, (3) environmental stewardship/planetary health, and (4) a statutory link between accredited education and professional regulation (Table 1). By articulating separate but related definitions of “nursing” and “a nurse,” ICN clarifies that the discipline is broader than any single role, while the professional title is reserved for those who meet specific educational and regulatory thresholds. This separation elevates nurse leadership by explicitly naming leadership, management, policy engagement, and systems-level responsibilities as part of what it means to be “a nurse,” thereby providing a normative basis for nurses to claim decision-making authority in health-system governance. At the same time, the requirement that a nurse be “educated in nursing science and regulated to practice” codifies licensure as a defining feature of the role, offering legislators and regulators in LMICs a clearer template for protected titles, scopes of practice, and accountability mechanisms. In resource-constrained settings, this dual move can either empower professional advocacy by strengthening the legal and policy case for advanced nursing roles or entrench hierarchy, depending on how inclusively governments integrate auxiliary and community-based providers into new regulatory pathways.

**Table 1.** Evolution of the ICN, Nursing, and “a Nurse” Definitions.

Year & source	Definition (abridged)	Notable shifts/emphases
(Henderson, 1966)	“The unique function of the nurse is to assist the individual […] and to help him gain independence as rapidly as possible.”	Individual focus Patient independence Bedside, curative orientation
(ICN, 2002)	“Nursing encompasses autonomous and collaborative care of individuals of all ages […] Advocacy, research, shaping health policy, health-systems management, and education are also key nursing roles.”	Expansion to families and communities Adds advocacy, research, policy, management, and education Introduces “autonomous and collaborative”
(White et al., 2025)—definition of “nursing”	“Nursing is a profession dedicated to upholding everyone's right to enjoy the highest attainable standard of health […] Grounded in science-based disciplinary knowledge, ethics, and technical capability, nursing is committed to compassion, social justice, and planetary well-being.”	Explicit human-rights framing Cultural safety and planetary health Links profession to accredited education and regulation Elevates systems leadership
(White et al., 2025)—definition of “a nurse”	“A nurse is a professional educated in nursing science and regulated to practice. Nurses enhance health literacy, promote health, prevent illness, protect safety, alleviate suffering, and work autonomously and collaboratively to improve health through advocacy, evidence-informed decision-making, and culturally safe, therapeutic relationships.”	Reserves the professional title for those meeting education + regulation thresholds (i.e., a protected title), while distinguishing the practitioner from the discipline. Provides a legal template for licensure statutes—scope of practice, accountability mechanisms, and title protection. Names leadership, management, and policy-shaping as intrinsic professional work, legitimizing nurses’ decision-making roles in health-system governance. Clarifies autonomy and delegation/supervision responsibilities, and affirms roles in disaster response and sustaining safe, environmentally responsible care.

### Process Strengths and Blind Spots

ICN used a five-step methodology—systematic literature synthesis, multilingual open survey, five-round Delphi with 21 experts, National Nursing Association consultation, and final board ratification—meeting modern norms for participatory policy design (White et al., 2025). Risk matrices, ethics protocols, and aggregated demographics are publicly available, offering a template other professions might emulate. Additional strengths include the combination of breadth and depth (multiregional, multilingual consultation paired with multiple Delphi rounds), explicit documentation of assumptions and risks, and the political legitimacy conferred by board and council-level endorsement (White et al., 2025). The report also notes representation from LMIC settings (e.g., Eswatini, the Philippines, Rwanda, and India) and Indigenous scholarship within the expert group, which strengthens cultural-safety claims and enhances relevance for diverse contexts (White et al., 2025).

Yet two blind spots matter for LMICs. First, only proportional—not granular—regional representation data are published, obscuring whether voices from the regions facing the largest projected nurse shortfalls (sub-Saharan Africa and South Asia) were fully weighted (ICN, 2025; WHO, 2022). The composition of the Delphi panel seems to be predominantly influenced by regulatory and academic elites, which may lead to an emphasis on well-funded practice frameworks at the expense of the challenges faced in resource-limited environments, particularly in rural contexts. These gaps suggest that country-level roll-outs should include local Delphi supplements, structured stakeholder hearings, or “road maps” co-created with frontline practitioners to ensure contextual validity.

### Three Paradigm Shifts Viewed From LMIC Settings

- **Planetary** Interdependence. Climate-vulnerable LMICs—responsible for <10 % of global emissions yet over 70 % of disaster mortality—could leverage nursing's new environmental mandate in national adaptation plans (United Nations Office for Disaster Risk Reduction, 2024; World Economic Forum, 2023). Integrating planetary-health competencies into crowded curricula will, however, require donor-supported faculty development and simulation resources (Ghimire & Ghimire, 2025).
- **Education–Regulation Coupling.** The 2025 ICN definitions reserve the protected title “nurse” for individuals educated in nursing science and regulated to practice in accordance with established standards and ethical codes (White et al., 2025). Furthermore, the document does not prescribe a specific institutional model, such as an independent nursing council or a ministry-based directorate. Instead, it delineates functional regulatory expectations that any model must fulfill, including: legal protection for the title “nurse,” enforceable standards for scope of practice and competency, mandatory registration on a publicly accessible register, due process for fitness-to-practice procedures, and the accreditation and inspection of educational programs with the authority to enforce compliance (White et al., 2025). In many LMICs, however, nursing councils remain under Ministries of Health, which can constrain autonomy and regulatory effectiveness, for example, limited resources and distance from frontline realities weaken standards enforcement (McGivern et al., 2024); dominance of medical professional bodies narrows nursing's policy influence (Putturaj & Prashanth, 2017); and hierarchical governance structures contribute to nurses’ exclusion from policy and clinical decision-making (Dunn, 2025). These dynamics echo broader findings that only about 53% of member states report fully autonomous nursing regulators (National Council of State Boards of Nursing, 2020) and align with country case studies from South Asia and sub-Saharan Africa, where regulation nested within ministries limits professional self-governance (Elison et al., 2015; Nyante et al., 2024). A practical approach for LMICs involves a phased strategy: legislate protected titles and minimum education standards; fund registration, inspection, and fitness-to-practice functions; and create bridging routes for auxiliary cadres to attain regulated status—regardless of whether the regulator is housed within a ministry or constituted as an arm's-length council.
- Systems Leadership. The 2025 ICN report underscores leadership deficits: fewer than one in four LMIC ministries of health employ a Government Chief Nursing Officer, and 72 % of surveyed nurses feel their expertise is under-utilized (ICN, 2025). By naming systems leadership as intrinsic to nursing, the definitions create a normative mandate; however, definitions are norm-setting instruments rather than implementation mechanisms. ICN does not assume responsibility for the development of posts or pipelines; this responsibility rests with national entities. These actors are tasked with converting the established definitions into actionable policy instruments, including civil service classifications, statutory role descriptions, regulations regarding board composition, accreditation standards, and designated budget allocations (Bandeali & Maita, 2023; Ludin, 2025). Concretely, this translation involves embedding the language into job descriptions (e.g., establishing or upgrading Government Chief Nursing Officer roles), linking promotion criteria to leadership competencies, funding executive fellowships, and using donor compacts to finance and sustain senior nursing posts. In short, the global definition supplies rationale and vocabulary, while local operationalization supplies authority, resources, and accountability; in the absence of this step, the leadership clause remains symbolic rather than structural.

## Critical Limitations and Opportunities Specific to LMICs

The ICN definitions report states that its concepts “must be contextualized to diverse political, cultural, and economic realities” (White et al., 2025). That caveat is crucial for LMICs, which shoulder an estimated 89% of the projected global nursing shortfall (WHO, 2022). In these settings, the report's transformative potential converges on three inter-locking domains—knowledge pluralism, financing, and workforce mobility—each capable of widening or closing equity gaps depending on national follow-through.

### Knowledge Pluralism and Cultural Safety

While the report positions “science-based disciplinary knowledge” as nursing's intellectual core, it also insists that “culturally grounded knowledge systems must be respected in the design of nursing services” (White et al., 2025). Tension arises where Indigenous or faith-based epistemologies guide care-seeking behavior. Ministries of health could operationalize this principle through reciprocal evidence panels—bodies in which community knowledge holders and academically trained nurses co-review clinical guidelines before national roll-out. Such “dual-validation” arrangements have improved maternity-service uptake in parts of South Asia (Nagesh et al., 2024) and are being piloted in Guatemala's intercultural obstetric units (Chaudhry et al., 2018). Embedding panels into regulatory statutes would translate cultural-safety rhetoric into enforceable governance while preserving scientific rigor.

### Financing the New Professional Standard—Toward a Gender-Equity Dividend

The report candidly notes that “varying levels of nursing regulation, education, and development across countries may create barriers to adoption” (White et al., 2025). Nowhere is that barrier felt more acutely than in financing; hence, upgrading all programs to the accredited, regulation-ready level implied by the definitions will raise costs in resource-constrained settings (Sommers & Rio, 2023). The report, echoing the *Triple Impact* analysis, frames nursing investment as a “triple dividend” for health, gender equity, and economic growth (All-Party Parliamentary Group, 2016). Complementary ICN economic briefs estimate a monetary return on investment (ROI) of two to four U.S. dollars for every dollar invested in nursing education (ICN, 2017, 2024). Positioning nursing education as development infrastructure—aligned with universal health coverage (UHC) and sustainable development goals (SDG) targets—would make it eligible for concessional loans or climate-adaptation funds, particularly where nurses lead disaster-risk reduction (Ghimire & Ghimire, 2025). One fiscally sustainable model is the study-serve compact, in which tuition is waived in exchange for a fixed service period in underserved areas, ensuring public ROI while avoiding gender-skewed debt burdens.

### Workforce Planning, Mobility, and Ethical Inclusion

The report lists workforce planning and mobility as its first global implication, arguing that a unified definition “enables more accurate planning and mutual recognition of qualifications” (White et al., 2025). Standardization can indeed facilitate emergency deployments and professional exchange; however, the definitions acknowledge this risk: a portable credential “may intensify outbound flows from countries already experiencing shortages” (White et al., 2025). However, this risk can be mitigated through emerging bilateral models that convert potential brain drain into mutually beneficial capacity building. For example, the 2024 Nepal–UK Memorandum of Understanding provides a contemporary framework that ties ethical recruitment to reinvestment in the sending country (Ghimire et al., 2024). To operationalize the ICN's definitions without eroding domestic staffing, LMIC governments should embed three safeguards into such mobility agreements: (1) cost-sharing for predeparture training, (2) earmarked remittances for faculty development, and (3) transparent data dashboards that track vacancies against migration approvals. Comparable agreements between the Philippines and Germany have reportedly increased advanced-practice training slots in the Philippines, suggesting that regulated mobility can be a catalyst rather than a threat (Rodriguez et al., 2025).

## Conclusions: Importance to the Nursing Profession in LMICs

The ICN's renewed definitions of “nursing” and “a nurse” are neither an automatic panacea for LMICs nor a document written exclusively for high-income realities; rather, they constitute a catalytic framework whose value for the global nursing community will be determined by how deftly governments, regulators, and professional bodies adapt their rights-based, science-anchored vision to local constraints and opportunities. If the definitions remain a verbatim import—raising educational thresholds without financing, privileging formal licensure while marginalizing auxiliary cadres, and easing outbound mobility without reciprocal investment—they risk reinforcing structural inequities within and between countries, with particularly acute consequences in LMICs. At country level, practical next steps include: establishing national multistakeholder taskforces to compare existing laws and curricula against the ICN definitions; revising “scope-of-practice” and “protected-title” legislation to incorporate the new language while creating bridging pathways for auxiliary and community-based providers; embedding cultural safety, planetary health, and systems-leadership competencies into accreditation standards; and aligning workforce data systems so that planning, migration agreements, and financing decisions can be monitored against clear nursing indicators. These efforts will be most effective when nursing leaders in LMICs and HICs work together—through ICN, WHO, and regional networks—to share tools, evaluate implementation, and advocate collectively for the resources needed to realize the definitions’ intent. By translating the language into culturally attuned public messaging, embedding tiered competency metrics into existing accountability systems, forging study-serve financing compacts, and negotiating circular-skills agreements that convert migration into shared capacity building, the definitions can elevate nursing's status, expand pathways for community-based providers, and align workforce strategy with UHC and SDG commitments. In this sense, the ICN report offers a common reference point for collective action, but its transformational promise will be realized only through deliberate, context-sensitive governance that turns global rhetoric into equitable local impact.
